# The Relevancy of Paracetamol and Breastfeeding Post Infant Vaccination: A Systematic Review

**DOI:** 10.3390/pharmacy6020027

**Published:** 2018-03-28

**Authors:** Nurain Suleiman, Siti Hadijah Shamsudin, Razman Mohd Rus, Samsul Draman, Mai Nurul Ashikin Taib

**Affiliations:** 1 Pharmaceutical Services Division, Johor State Health Department, 81200 Johor Bahru, Johor, Malaysia; 2 Kulliyyah of Pharmacy, International Islamic University Malaysia, 25710 Kuantan, Pahang, Malaysia;shadijah@iium.edu.my; 3 Kulliyyah of Medicine, International Islamic University Malaysia, 25710 Kuantan, Pahang, Malaysia; razman@iium.edu.my (R.M.R.); nurin@iium.edu.my (S.D.); 4 Ara Damansara Medical Centre, 40150 Shah Alam, Selangor, Malaysia; dr.mai.nurulashikin@ramsaysimedarbyhealth.com

**Keywords:** paracetamol, breastfeeding, post, childhood, prophylactic, immunization, vaccination

## Abstract

**Background:** Paracetamol may be used as an antipyretic agent for the treatment of fever, as well as an analgesic in the treatment of mild to moderate pain post-vaccination in infants. The use of paracetamol during fever may be or may not be recommended since it may alter the natural human body immune response, although it may reduce fever and fussiness. **Objectives:** The aims of this study are to describe the effectiveness of breastfeeding in reducing pain and paracetamol in reducing fever and pain post infant vaccination. **Methods:** Data sources and study selection was conducted by electronic searching of six databases. Manual reference checks of all articles on paracetamol and breastfeeding post infant vaccination published in the English language between 1978 and 2017. Two levels of screening were used on 9614 citations, which include screening of abstracts and titles followed by full text screening. The data synthesis were tabulated into study characteristics, quality, and effects. **Results:** Systematic review of breastfeeding included three studies from 9614 database searches found significant benefit from breastfeeding in pain scores and the duration of crying, as well as behavioural changes. None of the studies stated the detriment of breastfeeding before, during, and after immunization. Systematic review of paracetamol effectiveness included four studies from 1177 database searches found significant benefit from prophylaxis paracetamol in fever, one study found significant benefit from prophylaxis paracetamol in fussiness, and one study’s results were found to be not significant. Two studies on evaluating the safety of prophylactic paracetamol in 2009 found that antibody responses to several antigens were significantly reduced, and the other study in 1988 found that antibody titres to DTP bacteria of placebo and PCM did not differ significantly. **Conclusions:** The relevancy of giving paracetamol post all types of vaccination may be questionable. Breastfeeding before, during, and after immunization are recommended for pain reduction and are proven effective. Further research is required in deciding if paracetamol is to be of rational use following infant immunization.

## 1. Introduction

Paracetamol may be used as an antipyretic agent for the treatment of fever, as well as an analgesic in the treatment of mild to moderate pain on post vaccination in child [1]. Current recommendations of different guidelines [2,3,4] note the option to give paracetamol prophylaxis for childhood vaccinations, but neither promote nor discourage routine use of prophylaxis. The theoretical explanation on paracetamol is that it will inhibit the synthesis of prostaglandin in the hypothalamus, then inhibits the hypothalamic heat-regulating centre, and finally produces antipyeresis. It will also peripherally block pain impulse generation, thus producing analgesic effects [1]. These are the reasons why the use of paracetamol during fever may or may not be recommended since it may alter the natural human body immune response, although it may reduce pain.

The Medical News by The Lancet on 19 October 2009 stated that ‘paracetamol (also known as acetaminophen) to reduce fever after vaccination is likely to be counterproductive’. There is a study to prove that that the antibody geometric mean concentration (GMC) is significantly lower in the paracetamol group than in the control group [5,6]. In fact, some evidence showed that prophylactic administration of an antipyretic drug around the time of vaccination may lower antibody responses to some vaccines [7,8]. Additionally, the vaccine, itself, may not be effective if paracetamol is given at an early stage to prevent fever following immunization. It may cause fewer antibodies to be produced, thus, it is possible that the vaccine may not work well [7]. Thus, this may suggest not to use paracetamol post vaccination in infants since it may contradict the Worlds Health Organization’s Expanded Programme on Immunization’s main aim.

The reduction of fever and pain following infant immunization is a high priority for the international community. Older recommendations for fever and pain treatment need to be revised since treating fever at an early stage and pain following infant immunization by paracetamol may be questionable since it may cause the vaccine injected to be less effective. Evidence-based health policies and programmes aiming to reduce fever and pain following infant immunization need reliable and valid information. Effective interventions to improve overall infant health need targeted health and social policies that are informed by reliable and valid epidemiological data. This study, using a systematic review, aimed to estimate the effectiveness of paracetamol for fever and natural intervention (e.g., breastfeeding) for pain following infant vaccination. Interventions used in the studies of antipyretic property of paracetamol were placed in two intervention categories. There are administration of prophylactic paracetamol and administration of paracetamol during fever. Meanwhile, interventions used in the studies of the analgesic property of breastfeeding were placed in two categories: breastfeeding and held in mothers’ arms but not fed. The aim of this study is to determine the effectiveness of breastfeeding as an analgesic property, as well as the safety of paracetamol’s antipyeretic properties post infants vaccination, and to provide evidence-based recommendations for clinical practice.

## 2. Method

### 2.1. Search Strategies 

Medical, environmental, and scientific databases were search to identify primary studies of the effects of breastfeeding before, during, and after immunization, as well as the effects of antipyretic agent following infant immunization in order to capture as many relevant citations as possible. The electronic searches were supplemented by hand searching of six databases which were accessed through EzProxy for the Off Campus Access Online Database for the International Islamic University Malaysia (IIUM) Students and Staffs. The databases include the Ovid LWW Total Access Collection and Medline, CINAHL (Cumulative Index to Nursing and Allied Health Literature) Plus with Fulltext, Science Direct, Proquest Dissertations and Theses, Proquest Education Journal, and Proquest Health and Medical Complete. Additionally, manual reference checks were conducted of all articles on paracetamol and breastfeeding post childhood vaccination published in the English language between 1978 and 2017. Two levels of screening were used on 9614 citations. The keywords that were used included in Table A1.

The titles and abstracts of the articles were scanned by two reviewers (N.S. and S.H.S.). Articles selected by the reviewers were retrieved in full and assessed for eligibility by the two reviewers. The reviewers did not contact the authors to identify additional studies, but the reviewers referred to reference lists from the identified trials. The reviewers were not blinded to the authors or settings of the scanned articles. 

### 2.2. Study Selection: Inclusion Criteria

Only reports with information on infants (for this study defined as up to 1 year of age) were included. All randomized trials and cohort (non-randomized) studies that included a placebo or unexposed group were included for the determination of effectiveness. Trials of different designs, however, were handled separately. The effectiveness of breastfeeding as an analgesia and physical intervention of fever as antipyretic were reviewed for the immunization and/vaccination procedure only. All prospective studies that reported data on variables of noxious stimuli with behavioural, physiological, hormonal, and metabolic changes were included since infants respond to these variables. For the determination of safety, all prospective studies were included. Papers that have funding sources were also included in this study. 

### 2.3. Study Selection: Exclusion Criteria

Reviews, meta-analyses, editorials, commentary, or conference abstracts were excluded in this study. Meta-analysis was excluded in this study because it was not feasible due to extensive variation in study features and methodological quality [9].

### 2.4. Data Collection and Analysis

There were two reviewers in this study. The study from World Health Organization also included two reviewers for systematic review [10]. The first reviewer screened all titles and abstracts of papers identified by the literature search. The second reviewer handled duplicate screening on a random selection of found titles or abstracts. The disagreements were discussed between both reviewers. All studies that had been identified as potentially relevant were retrieved and read in full to determine the eligibility for inclusion.

Data extractions were conducted by using a pre-defined data extraction template. Data that were extracted included design characteristics, study population and country, sample size, sample selection, age of participants, the exposure and outcome measures and results. 

### 2.5. Primary Outcome

The primary outcome was pain and/fever following infant immunization. Examples of validated observational measures for pain were the Douleur Aigue du Nouveau-ne (DAN) Scale, Facial Pain Rating Scale, and Neonatal/Infant Pain Scale (NIPS), Children’s Hospital of Eastern Ontario Pain Scale (CHEOPS), and cry duration. Examples of observational measures for fever were babies’ fussiness and temperature reading 38 °C or greater.

### 2.6. Validity Assessment 

The included trials were not masked to the reviewers (N.S. and S.H.S.). The methodological quality of each study was assessed by two independent reviewers using the Crowe Critical Appraisal Tool (CCAT) [11] to investigate internal validity (the extent to which the information is probably free of bias) with the following attributes. The CCAT was developed based on a wide number of previous critical appraisal tools, general research methods theory and reporting guidelines [11]. The tool was validated and has undergone testing for reliability and validity [11]. The CCAT appraised papers included in the review in eight categories. This tool uses scoring system in which each category is scored from zero in which no evidence to five in which highest evidence. Total scores of each study are presented as a percentage. The average scores of reviewers were reported.

### 2.7. Data Abstraction 

Data from each eligible study were extracted individually on custom-made data collection forms (designed specifically for each intervention) by two (2) reviewers (N.S. or S.H.S.), and the results were compared. The reviewers resolved any disagreements through discussion. 

### 2.8. Study Characteristics

Characteristics of included studies as well as the country of being conducted were displayed in Table A2 (for effectiveness of breastfeeding) and Table A3 (for effectiveness of prophylactic paracetamol and its safety). This study included research published in 1987 onwards.

### 2.9. Data Synthesis

Data syntheses were tabulated into study characteristics, quality, and effects. The original review of summarizing the evidence from studies of variable design will provide details how the differences between study results were investigated and how they were summarized [12]. 

Authors of trials were not contacted for further details or provision of original data if the published report contained insufficient information. The study findings, as reported by the authors, were included in this review. 

The data in this research cannot be pooled due to insufficient data regarding odds ratios or relative risk, as well as confidence intervals in each study.

### 2.10. Secondary Outcomes

Local and adverse reactions following infant immunization was reviewed in the study of prophylactic paracetamol post infant vaccination.

## 3. Results

### 3.1. Effectiveness of Breastfeeding as an Analgesic Property for Pain Following Childhood Vaccination

#### 3.1.1. Study Descriptions

**Figure A1:**
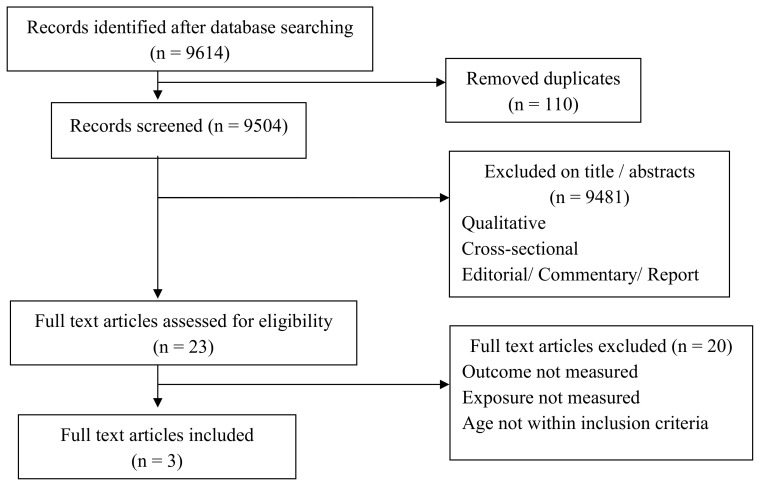
Flow diagramme of research strategy for effectiveness of breastfeeding as pain intervention.

Figure A1 presents a flow diagram of the search strategy. After duplicates were removed the search retrieved 9504 articles, of which 9481 are excluded (9400 on review of the abstracts/title and a further 81 after full-text paper assessment). Of the 23 reviewed full-text articles 19 were excluded because the outcome and exposure were not measured. Among these, one (1) was excluded because the age was not within the inclusion criteria. Finally, data from three (3) journal articles were included in the systematic review.

#### 3.1.2. Study Characteristics

Overall, there were three (3) studies that met the inclusion criteria and eligibility for study of the effectiveness of breastfeeding’s analgesic property for pain following immunization in infants. These studies were conducted mainly in the east coast country region, which include one (1) in Iran, one (1) in Jordan, and one (1) in Turkey. Studies began in 2007 and the latest study was in 2013.

These studies addressed two (2) of the intervention categories identified in the protocol: (i) breastfeeding; or (ii) held in mothers’ arms but not fed. All studies included babies not more than one (1) year of age. 

The researcher included randomized control trials and quasi-controlled trials that compared breastfeeding and combined interventions of interest with a placebo or control group for pain management during immunization in children aged from 0 months to 1 year of age. Among these, there were two (2) studies that were randomized controlled trials and only one (1) study that was a quasi-controlled trial. The primary outcome measure for pain was made by a health care worker or observer using observational methods; for example, the Douleur Aigue du Nouveau-ne (DAN) Scale, Facial Pain Rating Scale and Neonatal/Infant Pain Scale (NIPS), Children’s Hospital of Eastern Ontario Pain Scale (CHEOPS) and cry duration. However, all of these studies did not mention the duration of breastfeeding.

Among these three (3) studies, one (1) did not contain information about receiving approval by institutional review boards or ethics committees. On the other hand, two (2) of the three (3) studies mentioned that they obtained approval from institutional ethics review boards or committees. All of these studies mentioned that they obtained informed consent from the mothers. 

### 3.2. Methodological Quality of the Included Studies

The percentage of agreement on all key items for assessment of the methodological quality of the three (3) studies was from 75% to 83%; disagreements were resolved by consensus. Three (3) trials which include 316 infants aged zero (0) to 12 months examined the analgesic effects of breastfeeding.

### 3.3. Effects of Breastfeeding Post Infants Vaccination

In all three (3) studies, infants who were breastfeed before, during, and after procedure were compared with infants who were not breastfed. The level of pain was measured using cry duration [13,14], Neonatal Infant Pain Scale (NIPS) [14], Douleur Aigue du Nouveau-ne (DAN) Scale, Facial Pain Rating Scale (FPS) [14], Children’s Hospital of Eastern Ontarion Pain Scale (CHEOPS), as well as behavioural changes [13].

The reviews of all studies found significant benefit from breastfed in pain score and duration of crying, as well as behavioural changes. The pain score of one (1) study revealed a significant lower pain score in which *p* < 0.001 in the study by Razek et al., 2009 for the experimental group (breastfeeding group) than the control group (not breastfed). One study by Razek et al. in 2009 noted that the FPS for the intervention group represented “hurts little more” pain (38%) than the control group, which represented “hurts even more” (8.3%) Score 3 that indicate pain. Two (2) studies evaluated crying time and it was revealed that crying time was shorter in the intervention group rather than the control group [13,14]. Other than that, among two (2) studies that evaluated behavioural changes in heart rate and oxygen saturation, both were found to not differ significantly in mean heart rate elevation between control groups and experimental groups.

Breastfeeding was studied as an alternative to the painful procedure during immunization recently, with positive outcomes. Studies have demonstrated that breastfeeding [13,14,15], maternal holding [13], and skin to skin contact [13,14] statistically significantly reduced pain [15] and crying duration [13,14] in children following immunization. 

These studies showed that breastfeeding is effective as pain relief following immunization in infants.

### 3.4. Effectiveness of Prophylactic Paracteamol’s Antipyretic and Analgesic Properties and Its Safety for Fever Following Childhood Immunization 

#### 3.4.1. Study Descriptions

**Figure A2:**
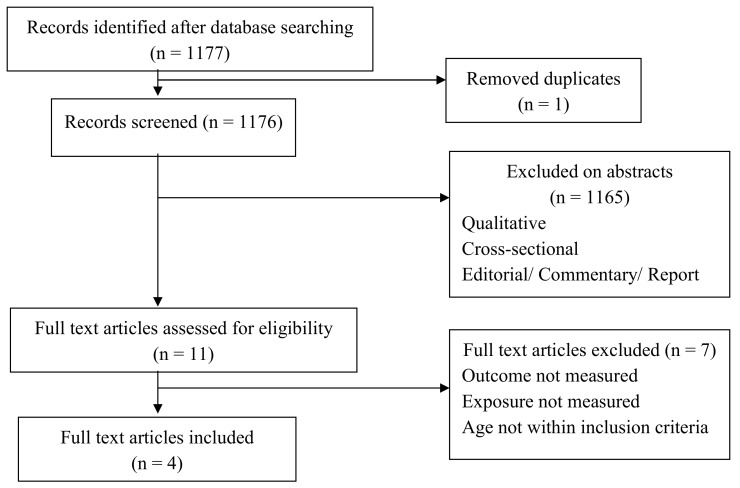
Flow diagram of research strategy for the effectiveness of prophylactic paracetamol for fever reduction post childhood vaccination.

Figure A2 presents a flow diagram of the search strategy. After duplicates were removed the search retrieved 1176 articles, of which 1165 were excluded (1100 on review of abstracts/title and a further 65 after full-text assessment). Of the 11 reviewed full-text articles two (2) were excluded because the outcome and exposure were not measured. Among these, five (5) were excluded because the ages were not within the inclusion criteria. Finally, data from four (4) journal articles were included in the systematic review.

#### 3.4.2. Study Characteristics

Overall, four (4) studies were assessed as being of sufficient quality to be included in the review. These studies were conducted mainly in Europe and east coast country regions, which include one (1) in the Czech Republic, one (1) in United States of America (USA), one (1) in Germany, and one (1) in Finland. Studies began in 1988 and the latest study was in 2013.

As mentioned before, these studies addressed two (2) intervention categories: (i) administration of prophylactic paracetamol; and (ii) non-prophylactic paracetamol for fever following childhood immunization.

All of these studies evaluated either the child was having fever or not [4,6,16,17], only one (1) study evaluated local systemic reactions [16], two (2) studies evaluated adverse reactions [16,17], and only one (1) study evaluated baby condition [4], as well as only two (2) studies evaluating the antibodies of children [6,17]. All studies included the age of babies from about six (6) weeks to around one (1) year of age [4,6,16,17]. All of these studies are also included in the systematic review. 

The researcher (N.S.) included all randomised controlled trials that compared prophylactic paracetamol use and/no prophylactic paracetamol use post infant vaccination. The primary outcome measure for fever was made by parents completing the diary and/questionnaires given by the researcher of the study.

Among these four (4) studies, three (3) of them mentioned that they obtained approval from institutional ethics review boards or committees [4,6,16]. All of these studies mentioned that they obtained informed consent from parents and/legal guardian, except the study by Uhari et al. (1988) did not mention they obtained consent from guardians, however, they had obtained ethical approval from the Medical Faculty of Oulu University.

### 3.5. Methodological Quality of the Included Studies

The percentage of agreement on all key items for assessment of the methodological quality of the four (4) studies ranged from 65% to 88%; disagreements were resolved by consensus. Four (4) trials which include 1156 infants aged zero (0) to 12 months of age examined the antipyretic effect of paracetamol. 

### 3.6. Effect of Prophylactic PCM for Fever and Pain Following Childhood Immunization

All studies compared children receiving prophylactic or non-prophylactic PCM post vaccination. Fever was measured using a body temperature ≥38 °C or >39.5 °C of axillary or rectal temperature. Meanwhile, baby condition was measured by the appearance of fussiness. 

The reviews of two (2) studies found significant benefit from paracetamol prophylaxis in fever [6,16] and only one (1) study found significant benefit from paracetamol prophylaxis in fussiness [4]. On the other hand, there was one (1) study that found a non-significant benefit from prophylaxis paracetamol in fever [17]. 

### 3.7. Safety of Prophylactic paracetamol Post Infant Vaccination

Other than that, there were two (2) studies that evaluated the safety of prophylactic paracetamol [6,17]. These studies revealed different outcomes, in which the study by Prymula et al. in 2009 found that antibody responses to several antigens were reduced significantly, and the other study by Uhari et al. in 1988 found that antibody titres to DTP bacteria of placebo and PCM did not differ significantly. The study by Prymula et al. in 2009 also noted that prophylactic paracetamol at the time of vaccination should not be routinely recommended, although febrile reactions were significantly reduced since antibody responses to several antigens were significantly reduced. 

Additionally, there was one (1) study by Jackson et al. in 2011 that was stopped because of the result of study by Prymula et al. in 2009. The study by Jackson et al. in 2011 also noted that the potential benefit of paracetamol prophylaxis in reducing the risk of fever and associated adverse events following contemporary infant immunizations appear to be outweighed by the potential harmful effects of paracetamol prophylaxis on vaccine immune responses. 

## 4. Discussion

Paracetamol was used as an antipyeretic agent and analgesic post vaccination in infants. However, its use seems questionable since, in theory, the use of paracetamol at early stages of fever may alter the vaccine function and cause the vaccine to be less effective [6]. Theoretically, the use of paracetamol may interfere natural body immune response by inhibiting prostaglandins (PGs), which are involved in the natural human body defence mechanisms. Most of the vaccines injected in children originate from attenuated organisms, which may cause infection. The organism might replicate over days or weeks, then result in immunity.

This study found that breastfeeding before, during, and after immunization reduced pain, as assessed using cry duration, DAN scale, FPS, NIPS, CHEOPS, and/or behavioural changes (heart rate and oxygen saturation). The proposed mechanisms of breastfeeding providing analgesia include (i) breastfeeding; and (ii) maternal holding and skin to skin contact [13]. 

The findings of the systemic review were consistent with the effectiveness of breastfeeding as an analgesic property in reducing pain of injection immunization in neonates [18]. Breastfeeding is a natural, cost-neutral, time-efficient, and convenient intervention that could be easily adopted from the perspectives of health care providers and parents [18]. Other than the nutritional and psychological value of breastfeeding, the analgesic properties may encourage more mothers to breastfeed [18]. 

The prophylactic antipyretic of paracetamol significantly reduced the febrile reactions of ≥38 °C after vaccinations. There were statistically significant differences in antibody responses between two groups which were lower in the prophylactic paracetamol group. One (1) recent study showed that there were significant reductions in the local and systemic symptoms in the prophylaxis group, but no significant difference between groups [16]. 

Only two (2) trials studied the antibody response [6,17], thus, the data cannot be pooled. Studies used different doses/schedules of antipyretic administration, and the age of participants or timing of administration were also markedly differed among studies. 

There were no studies that were identified in the literature search that evaluated the effectiveness of oral analgesics in which paracetamol for immunization pain [18]. Paediatricians may recommend oral analgesics to parents as a pain-relieving intervention for vaccine injection pain [18]. However, no evidence was found to recommend the use of either agent as a method of pain relief for vaccine injections. There were no studies that identified the paracetamol effects on vaccine injection pain, however, this agent was widely used. Thus, a study that addresses this issue may be warranted. 

## 5. Limitation

Methodological challenges and limitations of this review include the small number of studies for breastfeeding interventions, small sample size, limited age range of participants, limited number of vaccines evaluated, and variability in pain assessments. The included trials used various methods of assessing pain in infants, which made it difficult to combine and contrast the results.

## 6. Recommendation for Future Research

Further research is required in deciding paracetamol to be of rational use following infant immunization. 

Based on the researcher’s review, areas for future research were identified. The role of expressed breast milk has not been studied, and further research is needed. Finally, studies addressing whether the gap between research findings and clinical practice can be narrowed by communication and dissemination strategies aimed at practitioners, professional groups, and families will be important in establishing the common goal of pain-free, tolerable, and effective immunization for infants. 

Future trials should focus on the timing (before, with, or after) and route (oral or rectal) of administration of paracetamol, as well as on the subgroup of infants (term or preterm) for any correlation with the immune response. Future trials should focus on trials examining the prophylactic effect of paracetamol post vaccination antibody response since there was lack of studies regarding this issue. The mechanism underlying the reduction in immune/antibody response should also be explored. Trials should also be conducted in developing countries where over-the-counter use of antipyretics (including prophylactic) are common. Other confounding factors that might affect the antibody response, such as infant sleep post-immunization, should also be studied. 

## 7. Conclusions

The relevancy of giving, or the usage of, paracetamol post all types of vaccination is still questionable due to the safety issues this intervention might arise. 

From this systematic review, breastfeeding before, during, and after immunization were recommended for pain reduction and is proven effective.

The reviews showed that prophylactic antipyretic paracetamol administration leads to reduce of fever and fussiness. However, there was a reduction in antibody responses to some vaccine antigens. Future study and surveillance programs should also aim at assessing the effectiveness of programs where prophylactic paracetamol is given. The timing of administration of paracetamol should be discussed with the parents after explaining the benefits and risks.

## Appendix A

**Table A1:** Keywords for systematic review.

Database Searches	Items Measure	Keywords
Ovid LWW Total Access Collection and Medline, CINAHL Plus with Fulltext, Science Direct, Proquest Dissertations and Theses, Proquest Education Journal and Proquest Health and Medical Complete (data collected from published paper from 1987 until 2017)	(1) Pain (2) Breastfeeding	‘breastfeeding; pain or analgesia; following or post; immunization or vaccination; infant or newborn’
	(3) Fever and pain (4) paracetamol	‘feverish or febrile or fever; breastfeeding; temperature decrease; antipyretic; analgesic; following or post; immunization or vaccination; infant or newborn; antibody’

**Table A2:** Summary of relevant research on effectiveness of breastfeeding used as an intervention to decrease pain in infants.

No.	Author; Country; Year of Publication	Research Design	Study Population; Care Recipient % Boys; Care Recipient Age Mean (SD)	Sample Size: Baseline; Follow-Up	Exposure Measure	Outcome Measure	Quality Score (%)	Statistical Results	Conclusion
1	Modarres, Jazayeri, Rahnama, Montazeri, Iran, 2013 [Funding Source: Instituitional Review Board of the Tehran University of Medical Sciences]	True experiment: Placebo controlled trial	Full term neonates breastfed 2 minutes before, during and after Hepatitis B immunization or held in mothers’ arms but not fed; 83% boys; 39.4 (1.2) in control group and 39.1 (1.3) in experimental group weeks	130; 130; 130	Pain score measured using DAN scale (Facial expressions, limb movements and vocal expression)	Pain score	75	(1) Significant difference in mean of facial expressions of neonates between the control 2.58 (SD = 0.72) and experimental groups 1.39 (SD = 0.65). (*p* < 0.001). (2) Significant differences between two groups in mean of limb movements 1.92 (SD = 0.69) and experimental groups 0.83 (SD = 0.51). (*p* < 0.001) (3) Significant differences in mean of vocal expression between control 2.28 (SD = 0.57) and experimental groups 1.31 (SD = 0.68). (*p* < 0.001). (4) Significant difference in mean of Total DAN scores between control 6.78 (SD = 1.69) and experimental groups 3.52 (SD = 1.37). (*p* < 0.001)	Breastfeeding reduces pain and is effective way for pain relief during Hepatitis B injection
2.	Razek, El-Dein, Jordan, 2009 [Funding Source: None]	Quasi experiment: Counter balanced (cross-over)	Infants either breastfed or not; 64.2% boys; 1–12 months of age	120; 120; 120	(1) Pain score measured using Facial Pain Rating Scale before, during and after procedure (2) Duration of crying (3) Heart rates	(1) Pain rating scale (2) Crying time (3) Heart rate	75	(1) Significant difference in Facial Pain Rating Scale between control and experimental group (*p* < 0.05) (2) Significant difference in mean of Duration of Crying between control 148.66 s (SD 13.96) and experimental groups 125.33 s (SD 12.18). (*p* < 0.005) (3) Not differ significantly in mean of heart rate elevation between control group (before procedure 125.22 bpm SD 29.15, after procedure 162.25 bpm SD 40.22) and experimental group (before procedure 128.59 bpm SD 15.45, after procedure 149.210 bpm SD 20.510). *p* before procedure = 1.330, *p* after procedure=none	Breastfeeding and skin to skin contact significantly reduced the pain in infants receiving immunization. Pain Score also showed lesser in breastfeeding group.
3.	Efe, Ozer, Turkey, 2007 [Funding Source: Akdeniz University Scientific Research Project Unit]	True experiment: Placebo controlled trial	Healthy infants receiving 2nd, 3rd or 4th immunization of IM DTP either breastfed before, during and after injection or given not breastfed; 56.1% boys; 3.08 ± 1.32 months control, 2.79 ± 1.13 months breastfed	66; 66; 66	(1) Length of crying (2) Heart rate (3) Oxygen saturation levels	(1) Crying time (2) Behavioural changes	83	(1) Significant difference in mean of Crying duration between control 76.24 s (SD 49.61) and experimental 35.85 s (SD 40.11). *p* = 0.001 (2) Not differ significantly in mean of heart rate elevation between control group (during procedure 129.58 bpm SD 38.32, after procedure146.36 bpm SD 31.06) and experimental group (during procedure 138.85 bpm SD 35.89, after procedure153.36 bpm SD 29.60). *p* during procedure = 0.31, *p* after procedure = 0.352 (3) Not differ significantly in mean of oxygen saturation between control group (during procedure 95.85% SD 4.18, after procedure 95.33% SD 4.17) and experimental group (during procedure 96.64% SD 2.93, after procedure 95.97% SD 3.08). *p* during procedure = 0.379, *p* after procedure = 0.483	Breastfeeding, maternal holding, and skin to skin contact significantly reduced crying time in infants receiving immunization injection for DTP

**Table A3:** Summary of relevant research on effectiveness of prophylactic antipyretic used as an intervention to decrease fever in infants and its safety issue.

No.	Author; Country; Year of Publication	Research Design	Study Population; Care Recipient % Boys; Care Recipient Age Mean (SD)	Sample Size: Baseline; Follow-Up	Exposure Measure	Outcome Measure	Quality Score	Statistical Results	Conclusion
1.	Rose, Juergens, Schmoele-Thoma, Gruber, Baker; Germany; 2013 [Funding Source: Pfizer Inc.]	True experiment: Placebo controlled trial	Healthy infants who received three-dose infant series of PCV-7 and DTPa-HBV-IPV/Hib plus a toddler dose either received prophylactic paracetamol at vaccination and at 6–9 h interval thereafter or a control group that received no paracetamol; 51.5% boys; 2.4–11.7 months	301; 286; 245	(1) Incidence of fever (2) Baby Conditions (3) Crying	(1) Fever (2) Drowsiness (3) Decreased appetite (4) Decreased activity (5) Persistent inconsolable crying	83	(1) Significant difference in temperature ≥38 °C to ≤39 °C of control 35.8% and experimental 9.3% groups: → after dose 1 (*p* < 0.001) (2) Significant difference in temperature ≥38 °C to ≤39 °C of control 43.7% and experimental 19.7% groups: → after dose 2 (*p* = 0.000) (3) Significant difference in temperature ≥38 °C to ≤39 °C of control 45.6% and experimental 19.3% groups: → after dose 3 (*p* = 0.000) (4) No significant difference in temperature ≥38 °C to ≤39 °C of control 60% and experimental 51.5% groups: → after toddler dose (*p* = 0.221) (5) No significant difference in temperature ≥39 °C to ≤40 °C of control 4% and experimental 0% groups: → after dose 1 (*p* = 0.061) (6) No significant difference in temperature ≥39 °C to ≤40 °C of control 1.8% and experimental 0% groups: → after dose 2 (*p* = 0.238) (7) No significant difference in temperature ≥39 °C to ≤40 °C of control 1.9% and experimental 1.0% groups: → after dose 3 (*p* > 0.99) (8) No significant difference in temperature ≥39 °C to ≤40 °C of control 13.1% and experimental 4.6% groups: → after toddler dose (*p* = 0.072) (9) No significant difference in temperature >40 °C of control 1.1% and experimental 0% groups: → after toddler dose (*p* > 0.99) (10) Significant difference in drowsiness of control 64.7% and experimental 50.4% groups: → after dose 1 (*p* = 0.019) (11) No significant difference in drowsiness of control 58.3% and experimental 46.5% groups: → after dose 2 (*p* = 0.078) (12) No significant difference in drowsiness of control 45.6% and experimental 36.4% groups: → after dose 3 (*p* = 0.182) (13) No significant difference in drowsiness of control 50.4% and experimental 43.5% groups: → after toddler dose (*p* = 0.350) (14) No significant difference in decreased appetite of control 40% and experimental 30.3% groups: → after dose 1 (*p* = 0.118) (15) Significant difference in decreased appetite of control 42.7% and experimental 26.6% groups: → after dose 2 (*p* = 0.011) (16) No significant difference in decreased appetite of control 33.6% and experimental 23.0% groups: → after dose 3 (*p* = 0.101) (17) No significant difference in decreased appetite of control 45.2% and experimental 38.2% groups: → after toddler dose (*p* = 0.336) (18) No significant difference in decreased activity of control 46.3% and experimental 41.6% groups: → after dose 1 (*p* = 0.457) (19) Significant difference in decreased activity of control 48% and experimental 31% groups: → after dose 2 (*p* = 0.007) (20) Significant difference in decreased activity of control 40% and experimental 23.3% groups: → after dose 3 (*p* = 0.007) (21) Significant difference in decreased activity of control 48.3% and experimental 23.3% groups: → after toddler dose (*p* = 0.005) (22) Significant difference in persistent inconsolable crying of control 20% and experimental 9.5% groups: → after dose 1 (*p* = 0.031) (23) No significant difference in persistent inconsolable crying of control 15.8% and experimental 9.3% groups: → after dose 2 (*p* = 0.171) (24) No significant difference in persistent inconsolable crying of control 15.3% and experimental 14% groups: → after dose 3 (*p* = 0.849) (25) No significant difference in persistent inconsolable crying of control 17.1% and experimental 7.8% groups: → after toddler dose (*p* = 0.056)	(1) PCM reduced incidence of fever ≥38 °C, reduction significant in infants but not in toddler (2) Fever >39 °C was rare during infant series, thus, too few cases for assessment (3) PCM reduced incidence of drowsiness, reduction significant in infants after dose 1 but not in dose 2 and 3 also in toddler (4) PCM reduced incidence of decreased appetite, reduction significant in infants after dose 2, but not after dose 1 and 3 also in toddlers (5) PCM reduced incidence of decreased activity, reduction significant in infants after dose 2, 3, and in toddlers, but not after dose 1 (6) PCM reduced incidence of persistent inconsolable crying, reduction significant in infants after dose 1, but not in dose 2 and 3 also in toddlers
2.	Jackson, Peterson, Dunn, Hambidge, Dunstan, Starkovich, Yu, Benoit, Dominguez-Islas, Carste, Benson, Nelson; Czech Republic; 2011 [Funding Source: Centre for Disease Control and Preventive (CDC) through America’s Health Insurance Plans]	True experiment: Placebo controlled trial	Children received up to five PCM doses (10–15 mg/kg) or placebo following routine vaccinations; 51% boys; 31 weeks to 69 weeks	374; 352; 234	(1) Rectal temperature (2) Baby condition	(1) Fever (2) Fussiness (more than much more than usual and much more than usual)	83	(1) No significant difference in rectal temperature ≥38 °C between the control 22% and experimental groups 14% (*p* = 0.053) (2) No significant difference in rectal temperature ≥39 °C between the control 2% and experimental groups 0% (*p* = 0.08) (3) Significant difference in fussiness (more than much more than usual) between the control 62% and experimental groups 58% (*p* = 0.045) (4) Significant difference in fussiness (much more than usual) between the control 24% and experimental groups 10% (*p* = 0.001)	Acetaminophen may reduce risk of post-vaccination fussiness but not reduce fever
3.	Prymula, Siegrist, Chlibek, Zemlickova, Vackova, Smetana, Lommel, Kaliskova, Borys, Schuerman; Czech Republic; 2009 [Funding Source: GSK Biologicals]	True experiment: Placebo controlled trial	Children received 3 prophylactic PCM doses every 6 to 8 hours in first 24 h, or no prophylactic PCM after each vaccination with PHiD-CV co-administered with DTPa-HBV-IPV/Hib and oral human rotavirus vaccines; 51% boys; mean aged at time of 1st dose was 12.3 weeks (SD 2.13).	459; 459; 414	(1) Rectal temperature >39.5 °C after primary and after booster (2) Percentage of child with temperature ≥38 °C after at least one dose of prophylactic PCM after primary and after booster (3) Antibody GMC after primary and after boosting	(1) Fever (2) Antibody GMC	88	(1) Rectal temperature >39.5 °C was uncommon in both groups: → after primary: 1/226 participants (<1%) in prophylactic PCM group vs. 3/233 (1%) in no prophylactic group: → after booster: 3/178 (2%) vs. 2/172 (1%) (2) Percentage of child with temperature ≥38 °C after at least 1 dose of prophylactic PCM was significantly lower → after primary: 154/233 (66%) and → after booster: 64/178 (36%) in prophylactic PCM group than in no prophylactic PCM group: → after primary: 154/233 (66%) → after booster: 100/172 (58%) (3) Antibody GMC were significantly lower in prophylactic PCM group than in no prophylactic PCM group after primary vaccination for all ten pneumococcal vaccine serotypes, protein D, antipolyribosyl-ribitol phosphate, antidipthteria, antitetanus, and antipertactin.	Prophylactic administration of antipyretic drugs at time of vaccination should not routinely recommended, although febrile reactions significantly decreased since antibody responses to several antigens were reduced significantly
4.	Uhari, Hietala, Viljanen; Finland; 1988 [Funding Source: None]	True experiment: Placebo controlled trial	Healthy infants vaccinated with DTP or DTP-inactivated polio vaccine receive placebo or 75 mg PCM 4 h after vaccination; not mentioned; 5 months	295; 263; 263	(1) Temperature in the evening and the next morning (2) Percentages of temperature with no fever and fever in the evening and the next morning (3) Levels of IgG antibodies (for Diphtheria toxoid, Tetanus toxoid, Pertussis bacteria) (4) Frequency of fever during 24 h after DTP vaccination	(1) Fever (2) Antibody titres	65	(1) No significant difference in mean of temperature in the evening between the control 37.6 °C (SD 0.49) and experimental groups 37.6 °C (0.65). 95% confidence limits of the difference −0.1–0.1 (2) No significant difference in mean of temperature in the next morning between the control 37.6 °C (SD 0.53) and experimental groups 37.6 °C (0.53). 95% confidence limits of the difference −0.1–0.1 (3) No significant difference in mean percentages of temperature with no fever in the evening between the control 36.5% and experimental groups 37% (4) No significant difference in mean percentages of temperature with fever in the evening between the control 6.75% and experimental groups 6.75% (5) No significant difference in mean percentages of temperature with no fever in the next morning between the control 40% and experimental groups 35% (6) No significant difference in mean percentages of temperature with fever in the next morning between the control 5% and experimental groups 7.25% (7) No significant difference in mean levels of IgG antibodies (for Diphtheria toxoid) between the control 10.5 (SD = 6.3) and experimental groups 10.7 (SD = 6.6), 95% Confidence limits of differences −3.6–3.2 (8) No significant difference in mean levels of IgG antibodies (for Tetanus toxoid) between the control 16.6 (SD = 7.9) and experimental groups 14.2 (SD = 8.4), 95% Confidence limits of differences −1.9–6.7 (9) No significant difference in mean levels of IgG antibodies (for Pertusis bacteria) between the control 31.1 (SD = 20.0) and experimental groups 34.2 (SD = 25.3), 95% Confidence limits of differences −15.0–8.76 (10) No significant difference in frequency of fever during 24 h period after DTP vaccination between the control 48.5% and experimental groups 44.4%, 95% Confidence limits of differences −8.0–16.	Acetaminophen in a single dose schedule is ineffective in decreasing post-vaccination fever and antibody response also showed no significant difference in control and experimental groups

NS = Not significant; DTP = Diphtheria, Tetanus, and Pertussis; GMC = Geometric Mean Concentration.
